# Causal machine learning for healthcare and precision medicine

**DOI:** 10.1098/rsos.220638

**Published:** 2022-08-03

**Authors:** Pedro Sanchez, Jeremy P. Voisey, Tian Xia, Hannah I. Watson, Alison Q. O’Neil, Sotirios A. Tsaftaris

**Affiliations:** ^1^ School of Engineering, University of Edinburgh, Edinburgh, UK; ^2^ AI Research, Canon Medical Research Europe, Edinburgh, Lothian, UK

**Keywords:** causal machine learning, causal representation learning, precision medicine

## Abstract

Causal machine learning (CML) has experienced increasing popularity in healthcare. Beyond the inherent capabilities of adding domain knowledge into learning systems, CML provides a complete toolset for investigating how a system would react to an intervention (e.g. outcome given a treatment). Quantifying effects of interventions allows actionable decisions to be made while maintaining robustness in the presence of confounders. Here, we explore how causal inference can be incorporated into different aspects of clinical decision support systems by using recent advances in machine learning. Throughout this paper, we use Alzheimer’s disease to create examples for illustrating how CML can be advantageous in clinical scenarios. Furthermore, we discuss important challenges present in healthcare applications such as processing high-dimensional and unstructured data, generalization to out-of-distribution samples and temporal relationships, that despite the great effort from the research community remain to be solved. Finally, we review lines of research within causal representation learning, causal discovery and causal reasoning which offer the potential towards addressing the aforementioned challenges.

## 1. Introduction

Considerable progress has been made in predictive systems for healthcare following the advent of powerful machine learning (ML) approaches such as deep learning [1]. In healthcare, clinical decision support (CDS) tools make predictions for tasks such as detection, classification and/or segmentation from electronic health record (EHR) data such as medical images, clinical free-text notes, blood tests and genetic data. These systems are usually trained with supervised learning techniques. However, most CDS systems powered by ML techniques learn only associations between variables in the data, without distinguishing between causal relationships and (spurious) correlations.

CDS systems targeted at precision medicine (also known as personalized medicine) need to answer complex queries about how individuals would respond to interventions. A precision CDS system for Alzheimer’s disease (AD), for instance, should be able to *quantify* the effect of treating a patient with a given drug on the final outcome, e.g. predict the subsequent cognitive test score. Even with the appropriate data and perfect performance, current ML systems would predict the best treatment based only on previous correlations in data, which may not represent *actionable* information. Information is defined as *actionable* when it enables treatment (interventional) decisions to be based on a comparison between different scenarios (e.g. outcomes for treated versus not treated) for a given patient. Such systems need causal inference (CI) in order to make actionable and individualized treatment effect predictions [2].

A major upstream challenge in healthcare is how to acquire the necessary information to causally reason about treatments and outcomes. Modern healthcare data are multi-modal, high-dimensional and often unstructured. Information from medical images, genomics, clinical assessments and demographics must be taken into account when making predictions. A multi-modal approach better emulates how human experts use information to make predictions. In addition, many diseases are progressive over time, thus necessitating that time (the temporal dimension) is taken into account. Finally, any system must ensure that these predictions will be generalizable across deployment environments such as different hospitals, cities or countries.

Interestingly, it is the connection between CI and ML that can help alleviate these challenges. ML allows causal models to process high-dimensional and unstructured data by learning complex nonlinear relations between variables. CI adds an extra layer of understanding about a system with expert knowledge, which improves information merging from multi-modal data, generalization and explainability of current ML systems.

**Figure 1.**
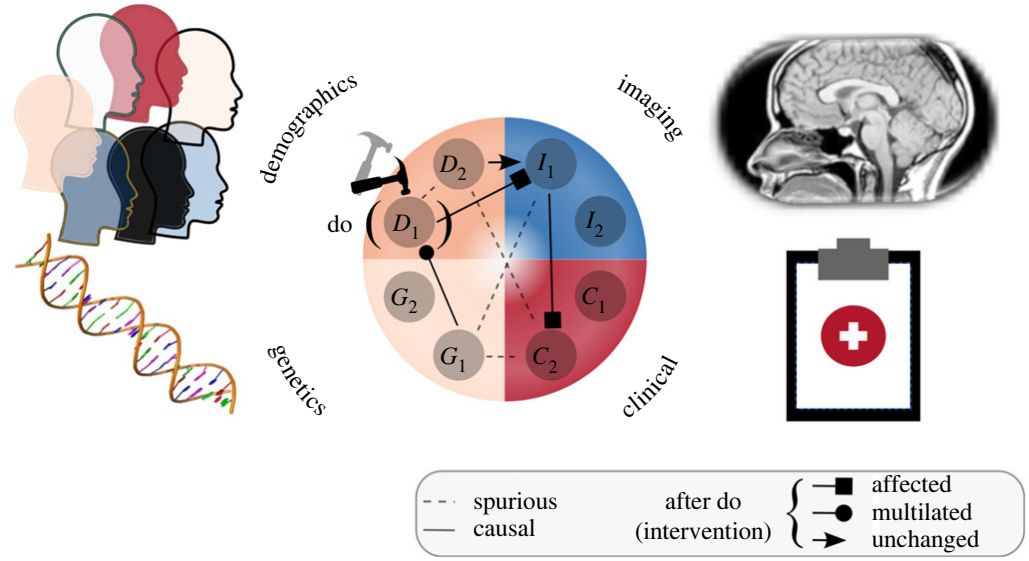
CML in healthcare helps understanding biases and formalizing reasoning about the effect of interventions. We illustrated, with a hypothetical example, that high-level features (causal *representations*) can be extracted from low-level data (e.g. *I*_1_ might correspond to the brain volume derived from a medical image) into a graph corresponding to the data generation process. CML can be used to *discover* which relationships between variables are spurious and which are causal, illustrated with lines dashed and solid lines respectively. Finally, CML offers tools for *reasoning* about the effect of interventions (shown with the *do*() operator). For instance, an intervention on *D*_1_ would only affect the downstream variables in the graph while other relationships are either not relevant (due to graph mutilation) or remain unchanged.

The *causal machine learning* (CML) literature offers several directions for addressing the aforementioned challenges when using observational data. Here, we categorize CML into three directions: (i) *Causal representation learning*—given high-dimensional data, learn to extract low-dimensional informative (causal) variables and their causal relations; (ii) *causal discovery*—given a set of variables, learn the causal relationships between them; and (iii) *causal reasoning*—given a set of variables and their causal relationships, analyse how a system will react to interventions. We illustrated in figure 1 how these CML directions can be incorporated into healthcare.

In this paper, we discuss how CML can improve personalized decision-making as well as help to mitigate pressing challenges in CDS systems. We review representative methods for CML, explaining how they can be used in a healthcare context. In particular, we (i) present the concept of causality and causal models; (ii) show how they can be useful in healthcare settings; (iii) discuss pressing challenges such as dealing with high-dimensional and unstructured data, out of distribution generalization and temporal information; and (iv) review potential research directions from CML.

## 2. What is causality?

We use a broad definition of causality: if *A* is a cause and *B* is an effect, then *B* relies on *A* for its value. As causal relations are directional, the reverse is not true; *A* does not rely on *B* for its value. The notion of *causality* thus enables analysis of how a system would respond to an *intervention*.

Questions such as ‘How will this disease progress if a patient is given treatment *X*?’ or ‘Would this patient still have experienced outcome *Z* if treatment *Y* was received?’ require methods from causality to understand how an intervention would affect a specific individual. In a clinical environment, causal reasoning can be useful for deciding which treatment will result in the best outcome. For instance, in an AD scenario, causality can answer queries such as ‘Which of drug *A* or drug *B* would best minimize the patient’s expected cognitive decline within a 5-year time span?’. Ideally, we would compare the outcomes of alternative treatments using observational (historical) data. However, the ‘fundamental problem of CI’ [3] is that for each unit (i.e. patient) we can observe either the result of treatment *A* or of treatment *B*, but never both at the same time. This is because after making a choice on a treatment, we cannot turn back time to undo the treatment. These queries that entertain hypothetical scenarios about individuals are called *potential outcomes*. Thus, we can observe only one of the potential consequences of an action; the unobserved quantity becomes a *counterfactual*. Causality’s mathematical formalism pioneered by Pearl [4] and Imbens and Rubin [5] allows these more challenging queries to be answered.

Most ML approaches are not (currently) able to identify cause and effect, because CI is fundamentally impossible to achieve without making assumptions [4,6]. Several of these assumptions can be satisfied through study design or external contextual knowledge, but none can be discovered solely from observational data.

Next, we introduce the reader to two ways of defining and reasoning about causal relationships: with structural causal models (SCMs) and with potential outcomes. We wrap up this section with an introduction to determining causal relationships, including the use of randomized controlled trials (RCT).

### 2.1. Structural causal models

The mathematical formalism around the so-called *do-calculus* and SCMs pioneered by the Turing Award winner Pearl [4] has allowed a graphical perspective to reasoning with data which heavily relies on domain knowledge. This formalism can model the data generation process and incorporate assumptions about a given problem. An intuitive and historical description of causality can be found in Pearl & Mackenzie's recent book *The Book of Why* [7].

An SCM $G\;:=(S,P_{N})$ consists of a collection **S** = (*f*_1_, …, *f*_*K*_) of structural assignments (called mechanisms)

2.1 $$X_{k}\;:=f_{k}({PA}_{k},N_{k}),$$

where **PA**_*k*_ is the set of parent variables of *X*_*k*_ (its direct causes) and *N*_*k*_ is a noise variable for modelling uncertainty. ***N*** = {*N*_1_, *N*_2_, …, *N*_*d*_} is also referred to as *exogenous* noise because it represents variables that were not included in the causal model, as opposed to the *endogenous* variables ***X*** = {*X*_1_, *X*_2_, …, *X*_*d*_} which are considered known or at least intended by design to be considered, and from which the set of parents **PA**_*k*_ are drawn. This model can be defined as a direct acyclic graph (DAG) in which the nodes are the variables and the edges are the causal mechanisms. One might consider other graphical structures which incorporate cycles and latent variables [8], depending on the nature of the data.

It is important to note that the causal mechanisms are representations of physical mechanisms that are present in the real world. Therefore, according to the principle of *independent causal mechanisms* (ICM), we assume that the causal generative process of a system’s variables is composed of autonomous modules that do not inform or influence each other [6,9]. This means that exogenous variables ***N*** are mutually independent with the following joint distribution $P(N)=\prod_{k=1}^{d}P(N_{k})$. Moreover, the joint distribution over the endogenous variables ***X*** can be factorized as a product of independent conditional mechanisms

2.2 $$P_{G}(X_{1},X_{2},\ldots ,X_{K})=\prod_{k=1}^{K}P_{G}(X_{k}|{PA}_{k}).$$

The causal framework now allows us to go beyond (i) associative predictions, and begin to answer (ii) interventional and (iii) counterfactual queries. These three tasks are also known as Pearl’s *causal hierarchy* [7]. The *do-calculus* introduces the notation do(*A*), to denote a system where we have *intervened* to fix the value of *A*. This allows us to sample from an interventional distribution $P_{X}^{G;\mathrm{do}(\cdots )}$, which has the advantage over an observational distribution $P_{X}^{G}$ that the causal structure enforces that only the descendants of the variable intervened upon will be modified by a given action. As illustrated in figure 1, after an intervention, the edges between the intervened variable and its parents are not relevant, resulting in a mutilated graph.

### 2.2. Potential outcomes

An alternative approach to CI is the *potential outcomes* framework proposed by Rubin [10]. In this framework, a response variable *Y* is used to measure the effect of some cause or treatment for a patient, *i*. The value of *Y* may be affected by the treatment assigned to *i*. To enable the treatment effect to be modelled, we represent the response with *two* variables $Y_{i}^{\left(0\right)}$ and $Y_{i}^{\left(1\right)}$ which denote ‘untreated’ and ‘treated’, respectively. The effect of the treatment on *i* is then the difference, $Y_{i}^{\left(1\right)}-Y_{i}^{\left(0\right)}$.

As a patient may *potentially* be untreated or treated, we refer to $Y_{i}^{\left(0\right)}$ and $Y_{i}^{\left(1\right)}$ as *potential outcomes*. It is, however, impossible to observe both simultaneously, according to the previously mentioned *fundamental problem of CI* [3]. This does not mean that CI itself is impossible, but it does bring challenges [5]. Causal reasoning in the potential outcome frameworks depends on obtaining an estimate for the joint probability distribution, *P*(*Y*^(0)^, *Y*^(1)^).

Both SCM and potential outcomes approaches have useful applications, and are used where appropriate throughout this article. In practice [11], while graphical SCMs are powerful for modelling assumption or identifying if an intervention is even possible or not, the potential outcomes literature is more focused on quantifying the effect of interventions. We note that single world intervention graphs [12] have been proposed as a way to unify them.

### 2.3. Determining cause and effect

Determining causal relationships often requires carefully designed experiments. There is a limit to how much can be learned using purely observational data.

The effects of causes can be determined through prospective experiments to observe an effect *E* after a cause *C* is tried or withheld, keeping constant all other possible factors. It is hard, and in most cases impossible, to control for all possible confounders of *C* and *E*. The gold standard for discovering a true causal effect is by performing an RCT, where the choice of *C* is randomized, thus removing confounding. For example, by randomly assigning a drug or a placebo to patients participating in an interventional study, we can measure the effect of the treatment, eliminating any bias that may have arisen in an observational study due to other confounding variables, such as lifestyle factors, that influence both the choice of using the drug and the impact of cognitive decline [13].

Note that the conditional probability *P*(*E*|*C*) of observing *E* after observing *C* can be different from the interventional probability *P*(*E*|do(*C*)) of observing *E* after doing/intervening on *C*. *P*(*E*|do(*C*)) means that only the descendants of *C* (in a causal graph) change after an intervention, all other variables maintain their values. In RCTs, ‘do’ is guaranteed and unconditioned, while with observational data such as historical EHRs, it is not, due to the presence of confounders.

Determining the causes of effects (the aetiology of diseases) requires hypotheses and experimentation where interventions are performed and studied to determine the necessary and sufficient conditions for an effect or disease to occur.

## 3. Why should we consider a causal framework in healthcare?

CI has made several contributions over the last few decades to fields such as social sciences, econometrics, epidemiology and aetiology [4,5], and it has recently spread to other healthcare fields such as medical imaging [14–16] and pharmacology [2]. In this section, we will elaborate on how causality can be used for improving medical decision-making.

Even though data from EHRs, for example, are usually observational, they have already been successfully leveraged in several ML applications [17], such as modelling disease progression [18], predicting disease deterioration [19] and discovering risk factors [20], as well as for predicting treatment responses [21]. Further, we now have evidence of algorithms which achieve superhuman performance in imaging tasks such as segmentation [22], detection of pathologies and classification [23]. However, predicting a disease with almost perfect accuracy for a given patient is not what precision medicine is trying to achieve [24]. Rather, we aim to build ML methods which extract *actionable* information from observational patient data in order to make interventional (treatment) decisions. This requires *CI*, which goes beyond standard supervised learning methods for prediction as detailed below.

In order to make actionable decisions at the patient level, one needs to estimate the treatment effect. The treatment effect is the *difference* between two potential outcomes: the *factual* outcome and the *counterfactual* outcome. For actionable predictions, we need algorithms that learn how to reason about hypothetical scenarios in which different actions could have been taken, creating, therefore, a decision boundary that can be navigated in order to improve patient outcome. There is recent evidence that humans use counterfactual reasoning to make causal judgements [25], lending support to this reasoning hypothesis.

This is what makes the problem of inferring treatment effect fundamentally different from standard supervised learning [2] as defined by the potential outcome framework [5,10]. When using observational datasets, by definition, we never observe the counterfactual outcome. Therefore, the best treatment for an individual—the main goal of precision medicine [26]—can only be identified with a model that is capable of causal reasoning as will be detailed in §3.3.

### 3.1. Alzheimer’s disease practical example

We now illustrate the notion of CML for healthcare with an example from *Alzheimer’s disease* (AD). A recent attempt to understand AD from a causal perspective [27,28] takes into account many biomarkers and uses domain knowledge (as opposed to RCTs) for deriving ground truth causal relationships. In this section, we present a simpler view with only three variables: chronological age,^1^ magnetic resonance (MR) images of the brain, and AD diagnosis. The diagnosis of AD is made by a clinician who takes into account all available clinical information, including images. We are particularly interested in MR images because analysing the relationship of high-dimensional data, such as medical images, is a task that can be more easily handled with ML techniques, the main focus of this paper.

**Figure 2.**
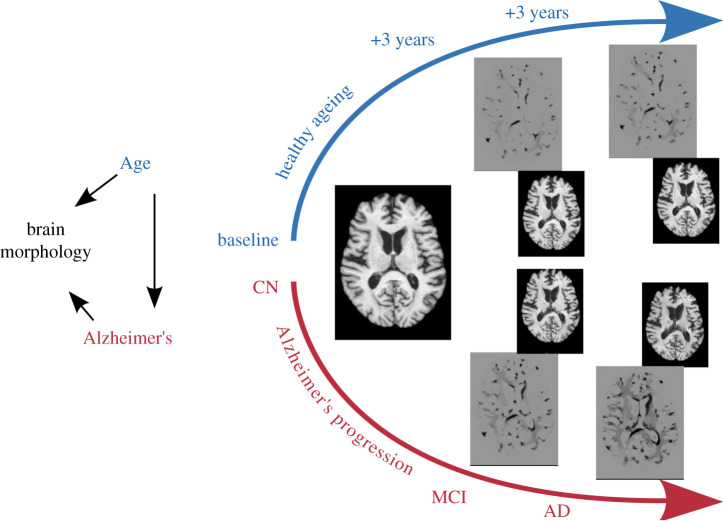
Causal graph (left) and illustration of how the brain changes in MR images in response to interventions on ‘Age’ or ‘Alzheimer’s disease status’. The images are axial slices of a brain MR scan. The middle image used as a baseline is from a patient aged 64 years old who is classified an cognitively normal (CN) within the Alzheimer’s Disease Neuroimaging Initiative (ADNI) database. All other images are synthesized with a conditional generative model [39]. The images with grey background are difference images obtained by subtracting the synthesized image from the baseline. The upper sequence of images is generated by fixing Alzheimer’s status at CN and increasing age by 3 years. The bottom images are generated by fixing the age at 64 and increasing Alzheimer’s status to MCI and AD, as discussed in the main text.

AD is a type of cognitive decline that generally appears later in life [30]. AD is associated with brain atrophy [31,32], i.e. volumetric reduction of grey matter. We consider that AD causes the symptom of brain morphology change, following Richens *et al.* [33], by arguing that a high-dimensional variable such as the MR image is caused by the factors that generated it; this modelling choice has been previously used in the causality literature [34–36]. Further, it is well established that atrophy also occurs during normal ageing [37,38]. Time does not depend on any biological variable, therefore chronological age cannot be caused by AD nor any change in brain morphology. In this scenario, we can assume that age is a confounder of brain morphology, measured by the MR image, and AD diagnosis. These relationships are illustrated in the causal graph in figure 2.

To model the effect of having age as a confounder of brain morphology and AD, we use a conditional generative model from Xia *et al.* [39],^2^ in which we condition on age and AD diagnosis for brain MRI image generation. We then synthesize images of a patient at different ages and with different AD status as depicted in figure 2. In particular, we control for (i.e. condition on) one variable while intervening on the other. That is, we synthesize images based on a patient who is cognitively normal (CN) for their age of 64 years. We then fix the Alzheimer’s status at CN and increase the age by 3 years for three steps, resulting in images of the same CN patient at ages 64, 67, 70, 73. At the same time, we synthesize images with different Alzheimer’s status by fixing the age at 64 and changing the Alzheimer’s status from mild cognitive impairment to a clinical diagnosis of AD.

This example illustrates the effect of *confounding bias*. By observing qualitatively the difference between the baseline and synthesized images, we see that ageing and AD have similar effects on the brain.^3^ That is, that both variables change the volume of brain when intervened on independently.

Throughout the paper, we will further add variables and causal links to this example to illustrate how healthcare problems can become more complex and how a causal approach might mitigate some of the main challenges. In particular, we will build on this example by explaining some consequences of causal modelling for dealing with high-dimensional and unstructured data, generalization and temporal information.

### 3.2. Modelling the data generation process

The AD example illustrates the importance of considering causal relationships in a ML scenario. Namely, causality gives the ability to model and identify types and sources of bias.^4^ To correctly identify which variables to control for (as means to mitigate confounding bias), causal diagrams [4] offer a direct means of visual exploration and consequently explanation [40,41].

Castro *et al.* [14] details further how understanding the causal generating process can be useful in medical imaging. By representing the variables of a particular problem and their causal relationships as a causal graph, one can model *domain shifts*, such as population shift (different cohorts), acquisition shift (different sites or scanners) and annotation shift (different annotators), and data scarcity (imbalanced classes). A benefit of reasoning causally about a problem domain is transparency, by offering a clear and precise language to communicate assumptions about the collected data [14,42,43]. In a similar vein, models whose architecture mirrors an assumed causal graph can be desirable in applications where interpretability is important [44].

In the AD setting above, a classifier naively trained to perform diagnosis from MR images of the brain might focus on the brain atrophy alone. This classifier may show reduced performance in younger adults with AD or for CN older adults, leading to potentially incorrect diagnosis. To illustrate this, we report the results of a convolutional neural network classifier trained and tested on the ADNI dataset following the same setting as Xia *et al.* [45].^5^ Table 1 shows that as feared, healthy older patients (80–90 years old) are less accurately predicted because ageing itself causes the brain to have Alzheimer’s-like patterns.

**Table 1.** Illustration of how a naively trained classifier (a neural network) fails when the data generation process and causal structure are not identified. We report the precision and recall on the test set when training a classifier for diagnosing AD. We stratify the results by age. We highlight that the group with worse performance is the older cognitively normal patients due to the confounding bias described in the main text. After training with counterfactually augmented data, the classifier’s precision for the worse performance age group improved. These results were replicated from our previous work Xia *et al.* [45].

age range (years)		60–70	70–80	80–90
naive	precision	87.7	91.4	75.5
	recall	92.5	94.2	97.1
counterfactually augmented	precision	88.3	93.6	84.2
	recall	91.5	96.5	95.7

Indeed, using augmented data based on causal knowledge is a solution discussed in Xia *et al.* [45], whereby the training data are augmented with counterfactual images of a patient when intervening on age. That is, images of a patient at different ages (while controlling for Alzheimer’s status) are synthesized so the classifier learns how to differentiate the effects of ageing versus AD in brain images.

This causal knowledge enables the formulation of best strategies for mitigating data bias(es) and improving generalization (further detailed in §4.3). For example, if after modelling the data distribution, an acquisition shift becomes apparent (e.g. training data were obtained with a specific MR sequence but the model will be evaluated on data from a different sequence), then data augmentation strategies can be designed to increase robustness of the learned representation. The acquisition shift—e.g. different intensities due to different scanners—might be modelled according to the physics of the (sensing) systems. Ultimately, creating a diagram of the data generation process helps rationalize/visualize which are the best strategies to solve the problem.

### 3.3. Treatment effect and precision medicine

Beyond diagnosis, a major challenge in healthcare is ascertaining whether a given treatment influences an outcome. For a binary treatment decision, for instance, the aim is to estimate the *average treatment effect* (ATE), *E*[*Y*^(1)^ − *Y*^(0)^], where *Y*^(1)^ is the outcome given the treatment and *Y*^(0)^ is the outcome without it (control). As it is impossible to observe both potential outcomes *Y*^(0)^ and $Y_{i}^{\left(1\right)}$ for a given patient *i*, this is typically estimated using *E*[*Y*|*T* = 1] − *E*[*Y*|*T* = 0], where *T* is the treatment assignment.

The treatment assignment and outcomes, however, both depend on the patient’s condition in normal clinical conditions. This results in confounding, which is best mitigated by the use of an RCT (§2.3). Performing an RCT as detailed in §2.3, however, is not always feasible, and CI techniques can be used to estimate the causal effect of treatment from observational data [46]. A number of assumptions need to hold in order for the treatment effect to be identifiable from observational data [5,47]. Conditional exchangeability (ignorability) assumes there are no unmeasured confounders. Positivity (overlap) is the assumption that every patient has a chance of receiving each treatment. Consistency assumes that the treatment is defined unambiguously. Continuing the Alzheimer’s example, Charpignon *et al.* [48] explore drug re-purposing by emulating an RCT with a target trial [49] and find indications that metformin (a drug classically used for diabetes) might prevent dementia.

**Figure 3.**
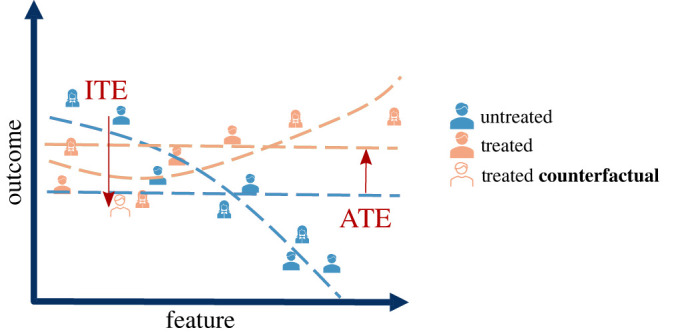
We illustrate the difference between individualized and average treatment effect (ITE versus ATE). ‘Feature’ represents patient characteristics, which would be multi-dimensional in reality. ‘Outcome’ is some measure of response to the treatment, where a more positive value is preferable. The ITE for each patient is the difference between actual and the counterfactual outcome. We show an example counterfactual to highlight that ITE for some patients might differ from the average (ATE). By employing causal inference methods to estimate individualized treatment effects, we can understand which patients benefit from certain medication and which patients do not, thus enabling us to make personalized treatment recommendations. Note that the patient data points are evenly distributed along the feature axis, which would indicate that this data comes from an RCT (due to lack of bias). The estimation of treatment affect using observational data is subject to confounding as patient characteristics affect both the selection of treatment and outcome. Causal inference methods need to mitigate this.

Note that even if the treatment effect is estimated using data from a well-designed RCT, *E*[*Y*| *T* = 1] − *E*[*Y*| *T* = 0] is the *average* treatment effect across the study population. However, there is evidence [2] that for any given treatment, it is likely that only a small proportion of subjects will actually respond in a manner that resembles the ‘average’ patient, as illustrated in figure 3. In other words, the treatment effect can be highly heterogeneous across a population. The aim of *precision medicine* is to determine the best treatment for an *individual* [24], rather than simply measuring the average response across a population. In order to answer this question for a binary treatment decision, it is necessary to estimate *τ*_*i*_ = *Y*_*i*_(1) − *Y*_*i*_(0) for a patient *i*. This is known as the individualized treatment effect. As this estimation is performed using a conditional average, this is also referred to as the conditional average treatment effect (CATE) [50].

A long-term goal of precision medicine [2] includes personalized risk assessment and prevention. Without a causal model to distinguish these questions from simpler prediction systems, interpretational mistakes will arise. In order to design more robust and effective ML methods for personalized treatment recommendations, it is vital that we gain a deeper theoretical understanding of the challenges and limitations of modelling multiple treatment options, combinations and treatment dosages from observational data.

## 4. Causal machine learning for complex data

In §3, we focused on causal reasoning in situations where the causal models are known (at least partially) and variables are well demarcated. We refer the reader to Bica *et al.* [2] for a comprehensive review on these methods. Most healthcare problems, however, have challenges that are upstream of causal reasoning. In this section, we highlight the need to deal with high-dimensional and multi-modal data as well as with temporal information and discuss generalization in out-of-distribution settings when learning from unstructured data.

### 4.1. Multi-modal data

AD, in common with other major diseases such as diabetes and cancer, has multiple causes arising from complex interactions between genetic and environmental factors. Indeed, a recent attempt [27] to build causal graphs for describing AD takes into account data derived from several data sources and modalities, including patient demographics, clinical measurements, genetic data and imaging exams. Uleman *et al.* [28], in particular, creates a causal graph^6^ with clusters of nodes related to brain health, physical health and psychosocial health, illustrating the complexity of AD.

The above example illustrates that modern healthcare is multi-modal. New ways of measuring biomarkers are increasingly accessible and affordable, but integrating this information is not trivial. Information from different sources needs to be transformed to a space where information can be combined, and the common information across modalities needs to be disentangled from the unique information within each modality [52]. This is critical for developing CDS systems capable of integrating images, text and genomics data. In addition, performing interventions [53] with complex data representations and functions is challenging. Strategies for counterfactual prediction [4] are simpler with scalar variables and linear functions. Interventions can have qualitatively distinct behaviours and should be understood as acting on high-level features rather than purely on the raw data.

On the other hand, the availability of more variables might mean that some assumptions which are made in classical CI are more realistic. In particular, most methods consider the assumption of *conditional exchangeability* (or causal *sufficiency* [54]), as in §3.3. In practice, the conditional exchangeability assumption may often not be true due to the presence of unmeasured confounders. However, observing more variables might reduce the probability of this, rendering the assumption more plausible.

### 4.2. Temporal data

It is well known that a gene called apolipoprotein *E* is associated with an increased risk of AD [55,56]. However, environmental factors, such as education [57–59], also have an impact on dementia. In other words, environmental factors over time contribute to different disease trajectories in AD. In addition, there are possible loops in the causal diagram [28]. Wang & Holtzman [60] illustrate, for instance, a positive feedback loop between sleep and AD. That is, poor sleep quality aggravates amyloid-beta and tau pathology concentrations, potentially leading to neuronal dysfunction, which, in turn, leads to worse sleep quality. It is, therefore, important to consider data-driven approaches for understanding and modelling the progression of disease over time [61].

At the same time, using temporal information for inferring causation can be traced back to one of the first definitions of causality by Hume [62]. Quoting Hume [62]: ‘we may define a cause to be an object followed by another, and where all the objects, similar to the first, are followed by objects similar to the second’. There are many strategies for incorporating time into causal models since using SCMs with directed acyclic graphs (as defined in §2.1) is not enough in this context. A classical model of causality for time series developed by Granger [63] considers *X* → *Y* if past *X* is predictive of future *Y*. Therefore, inferring causality from time-series data is at the core of CML. Bongers *et al.* [8] show that SCMs can be defined with latent variables and cycles, allowing temporal relationships. Early work has used temporal CI in neuroscience [64], but the application of temporal CI in combination with ML for understanding and dealing with complex disease remains largely unexplored.

Managing diseases such as AD can be challenging due to the heterogeneity of symptoms and their trajectory over time across the population. A pathology might evolve differently for patients with different covariates. For treatment decisions in a longitudinal setting, CI methods need to model patient history and treatment timing [65]. Estimating trajectories under different possible future treatment plans (interventions) is extremely important [66]. CDS systems need to take into account the current health state of the patient, to make predictions about the potential outcomes for hypothetical future treatment plans, to enable decision-makers to choose the sequence and timing of treatments that will lead to the best patient outcome [66–68].

### 4.3. Out-of-distribution generalization with unstructured and high-dimensional data

The challenge of integrating different modalities and temporal information increases when unstructured data is used. Most causality theory was originally developed in the context of epidemiology, econometrics, social sciences and other fields wherein the variables of interest tend to be scalars [4,5]. In healthcare, however, the use of imaging exams and free-text reports poses significant challenges for consistent and robust extraction of meaningful information. The processing of unstructured data is mostly tackled with ML, and *generalization* is one of the biggest challenges for learning algorithms.

In its most basic form, generalization is the ability to correctly categorize new samples that differ from those used for training [69]. However, when learning from data, the notion of generalization has many facets. Here, we are interested in a realistic setting where the test data distribution might be different from the training data distribution. This setting is often referred to as *out-of-distribution generalization*. Distribution shifts are often caused by a change in environment (e.g. different hospitals). We wish to present a causal perspective [70–72] on generalization which unifies many ML settings. Causal relationships are stable across different environments [73]. In a causal learning, the prediction should be invariant to distribution shifts [74].

As the use of ML in high-impact domains becomes widespread, the importance of evaluating safety has increased. A key aspect is evaluating how robust a model is to changes in environment (or domain), which typically requires applying the model to multiple independent datasets [75]. Since the cost of collecting such datasets is often prohibitive, CI argues that providing structure (which comes from expert knowledge) is essential for increasing robustness in real life [4].

**Figure 4.**
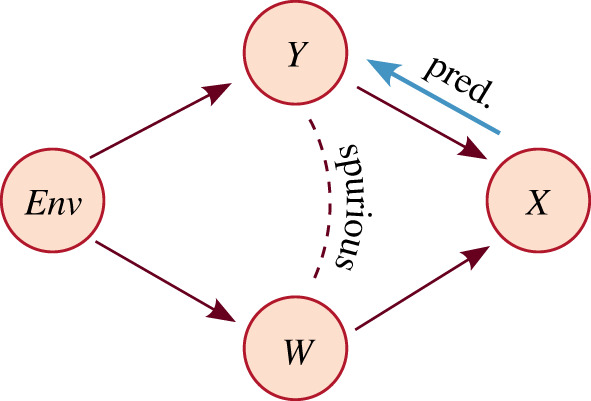
Reasoning about generalization of a prediction task with a causal graph. Anti-causal prediction and a spurious association that may lead to shortcut learning are illustrated.

Imagine a prediction problem where the goal is to learn *P*(*Y*|*X*), with the causal graph illustrated in figure 4. We consider an environment variable *Env* which controls the relationship between *Y* and *W*. *Env* is a confounder *Y* ← *Env* → *W* and *X* is caused by the two variables *Y* → *X* ← *W*.

Firstly, we consider the view that most prediction problems are in the anti-causal direction [34–36,76].^7^ That is, when making a prediction from a high-dimensional, unstructured variable *X* (e.g. a brain image) one is usually interested in extracting and/or categorizing one of its true generating factors *Y* (e.g. grey matter volume). *P*(*X*|*Y*), which represents the causal mechanism, *Y* → *X*, is independent of *P*(*Y*|*Env*); however, *P*(*Y*|*X*) is not, as *P*(*Y*|*X*) = *P*(*X*|*Y*)*P*(*Y*|*Env*)/*P*(*X*). Thus *P*(*Y*|*X*) changes as the environment changes.

Secondly, another (or many others) generating factor *W* is often correlated with *Y*, which might cause the predictor to learn the relationship between *X* and *W* instead of the *P*(*Y*|*X*). This is known as shortcut learning [79] as it may be easier to learn the *spurious correlation* than the required relationship. For example, suppose an imaging dataset *X* is collected from two hospitals, Env_1_ and Env_2_. Hospital Env_1_ has a large neurological disorder unit, hence a higher prevalence of AD status (denoted by *Y*), and uses a 3*T* MRI scanner (scanner type denoted by *W*). Hospital Env_2_ with no specialist unit, hence a lower prevalence of AD, happens to use a more common 1.5*T* MRI scanner. The model will learn the spurious correlation between *W* (scanner type) and *Y* (AD status).

We can now describe several ML settings based on this causal perspective by comparing data availability at train and test time. Classical *supervised learning* (or empirical risk minimization [80]) uses the strong assumption that the data from train and test sets are *independent and identically distributed (i.i.d.)*, therefore we assign the same environment for both sets. *Semi-supervised learning* [81] is a case where part of the training samples are not paired to annotations. *Continual (or Lifelong) learning* considers the case where data from different environments are added after training, and the challenge is to learn new environments without forgetting what has initially been learned. In *domain adaptation*, only unpaired data from the test environment is available during training. *Domain generalization* aims at learning how to become invariant to changes of environment, such that a new (unseen in training data) environment can be used for the test set. Enforcing *fairness* is important when *W* is a sensitive variable and the train set has *Y* and *W* spuriously^8^ correlated due to a choice of environment. Finally, learning from *imbalanced* datasets can be seen under this causal framework when a specific *Y* = *y* have different numbers of samples because of the environment, but the test environment might contain the same bias towards a specific value of *Y*.

## 5. Research directions in causal machine learning

Having discussed the utility of CML for healthcare including complex multimodal, temporal and unstructured data, the final section of this paper discusses some future research directions. We discuss CML according to the three categories defined in §1: (i) causal representation learning; (ii) causal discovery; and (iii) causal reasoning.

### 5.1. Causal representations

Representation learning [82] refers to a *compositional* view of ML. Instead of a mapping between input and output domains, we consider an intermediate representation that captures concepts about the world. This notion is essential when considering learning and reasoning with real healthcare data. High-dimensional and unstructured data, as considered in §4.3, are not organized in units that can be directly used in current causal models. In most situations, the variable of interest is not, for instance, the image itself, but one of its generating factors, for instance grey matter volume in the AD example.

*Causal* representation learning [9] extends the notion of learning factors about the world to modelling the relationships between variables with causal models. In other words, the goal is to model the representation domain Z as an SCM as in §2.1. Causal representation learning builds on top of the *disentangled* representation learning literature [83–85] towards enforcing stronger inductive bias as opposed to assumptions of factor independence commonly pursued by disentangled representations. The idea is to reinforce a hierarchy of latent variables following the causal model, which in turn should follow the real data generation process.

### 5.2. Causal discovery

Performing RCTs is very expensive and sometimes unethical or even impossible. For instance, to understand the impact of smoking in lung cancer, it would be necessary to force random individuals to smoke or not smoke. Most real data are observational and discovering causal relationships between the variables is more challenging. Considering a setting where the causal variables are **known**, *causal discovery* is the task of learning the direction of causal relationships between the variables. In some settings, we have many input variables and the goal is to construct the graph structure that best describes the data generation process.

Extensive background has been developed over the last three decades around discovering causal structures from observational data, as described in recent reviews of the subject [6,86–88]. Most methods rely on conditional independence tests, combinatorial exploration over possible DAGs and/or assumptions about the data generation process’s function class and noise distribution (e.g. the true causal relationships assumed to be linear, with additive noise, or that the exogenous noise has a Gaussian distribution) for finding the causal relations of given causal variables. In healthcare, Huang *et al.* [89] and Sanchez-Romero *et al.* [90] use causal discovery for learning how different physiological processes in the brain causally influence each other using functional MRI data.

Causal discovery is still an open area of research, and some of the major challenges in discovering causal effects [6,91] from observational data are the inability to (i) identify all potential sources of bias (unobserved confounders); (ii) select an appropriate functional form for all variables (model misspecification); and (iii) model temporal causal relationships.

### 5.3. Causal reasoning

It has been conjectured that humans internally build generative causal models for imagining approximate physical mechanisms through intuitive theories [35]. Similarly, the development of models that leverage the power of causal models around interventions would be useful. The causal models can be formally manipulated for measuring the effects of interventions. Using causal models for quantifying the effect of interventions and pondering about the best decision is known as *causal reasoning*. As previously discussed in §3.3, one of the key benefits from causal reasoning in healthcare is around personalized decision-making.

In SCMs (§2.1), personalized decision-making usually refers to the ability to answer counterfactual queries [53] about historical situations, such as ‘What would have happened if the patient had received alternative treatment *X*?’. Counterfactuals can be estimated with (i) a three-step procedure [53] (*abduction–action–prediction*) which has been recently enhanced with deep learning [15,92] using generative models such as normalizing flows [93], variational autoencoders [94] and diffusion probabilistic models [95] or (ii) *twin networks* [96] which augment the original SCM resulting in both factual and counterfactual variables represented simultaneously. Deep twin networks [97] leverage neural networks to further improve flexibility of the causal mechanisms. We note that quantifying the effect of interventions usually assumes that causal models are given either explicitly [15,98] or learned via causal discovery [99]. Aglietti *et al.* [98] evaluate their method with using a model of the causal effect of statin drugs on the levels of prostate specific antigen [100] while Pawlowski *et al.* [15] and Wang *et al.* [101] model the data generation process of the MRI images of the brain. Reinhold *et al.* [102] extend Pawlowski *et al.* [15] by adding pathological information about multiple sclerosis lesions.

In the potential outcomes framework (§2.2), a number of approaches have been proposed to estimate personalized (also called individualized or conditional average) treatment effect from observational data. These techniques include Bayesian additive regression trees [103], double ML [104,105], regularization of neural networks with integral probability metrics [106] or orthogonality constraints [107], Gaussian processes [108], generative adversarial networks [109] or energy-based models [110]. Another trend for estimating CATE are based on meta-learners [111,112]. In the meta-learning setting, traditional (supervised) ML is used to predict the conditional expectations of the potential outcomes and propensity. Then, CATE is computed by taking the difference between the estimated potential outcomes [112] or using a two-step procedure with regression adjustment, propensity weighting or doubly robust learning [111].

## 6. Conclusion

We have described the importance of considering CML in healthcare systems. We highlighted the need to design systems that take into account the data generation process. A causal perspective on ML contributes to the goal of building systems that are not just performing better (e.g. achiever higher accuracy), but are able to reason about potential effects of interventions at population and individual levels, closing the gap towards realizing precision medicine.

We have discussed key pressing challenges in precision medicine and healthcare, namely, using multi-modal, high-dimensional and unstructured data to make decisions that are generalizable across environments and take into account temporal information. We finally proposed opportunities drawing inspiration from causal representation learning, causal discovery and causal reasoning towards addressing these challenges.

## Data Availability

Data and relevant code for this research work are stored in GitHub: https://github.com/vios-s/BrainAgeing and have been archived within the Zenodo repository: https://doi.org/10.5281/zenodo.6832777 [113].

## Footnotes

1 Age can otherwise be measured in biological terms using, for instance, DNA methylation [29].

2 We take the model from Xia *et al.* [39] and run new demonstrative experiments for illustration in this paper.

3 See Xia *et al.* [39] for quantitative results confirming this hypothesis.

4 We refer to https://catalogofbias.org/biases for a catalogue of bias types.

5 Although we replicate results from Xia *et al.* [45], this work does not constitute an extension of the original paper. Rather, we use Xia *et al.* [45] as an example that illustrates how causality might impact standard machine learning.

6 Interestingly, Uleman *et al.* [28] gather expert knowledge using a group model-building technique [51] where multiple experts with complementary skills create a graph based on their combined mental models and assumptions.

7 We note that other seminal works [77,78] consider prediction a causal task because prediction should copy a cognitive human process of generating labels given the data.

8 We use the term *spurious* for features that correlate but do not have a causal relationship between each other.
